# P20 Evaluation of cefepime/enmetazobactam against MDR Enterobacterales

**DOI:** 10.1093/jacamr/dlae217.024

**Published:** 2025-01-23

**Authors:** J R Richards, M Wooton, J R Ward

**Affiliations:** Public Heath Wales, Cardiff, UK; Public Heath Wales, Cardiff, UK; Advanz Pharma, London, UK

## Abstract

**Background:**

Cefepime/enmetazobactam is a fourth generation cephalosporin in combination with a novel penicillanic acid sulfone ESBL inhibitor. Cefepime is stable to AmpC β-lactamases (class C) and OXA-48-like carbapenemases (class D) whilst enmetazobactam has activity against most class A β-lactamases (TEM, SHV, CTX-M). Limited activity has been reported against KPC carbapenemases and no activity seen against metallo-β-lactamases (class B). Cefepime/enmetazobactam is approved for use as treatment for complicated urinary tract infections (cUTI), pyelonephritis, hospital acquired pneumonia (HAP) and ventilator acquired pneumonia (VAP). The aim of this study was to understand the activity of cefepime/enmetazobactam against UK isolates of MDR Enterobacterales.

**Materials and methods:**

Cefepime/enmetazobactam MICs were determined on 203 clinical Enterobacterales using broth micro dilution (BMD) according to International Standard 20776-1. All isolates were resistant third generation cephalosporin and/or carbapenems, due to native and plasmid-mediated AmpC β-lactamases, ESBLs, OXA-48-like carbapenemases and efflux mechanisms. Ceftazidime-avibactam was tested concomitantly. Clinically derived susceptibility testing data for carbapenems, cephalosporins and aminoglycosides was also collected. EUCAST v14 clinical breakpoints were used to interpret MICs.

**Results:**

Overall cefepime/enmetazobactam susceptibility was 94.1% and resistance 5.9%. Cefepime/enmetazobactam susceptibility for third generation cephalosporin resistant Enterobacterales, harbouring either a CTX-M ESBL, hyper-expressed native (intrinsic) AmpC β-lactamase or plasmid-mediated AmpC β-lactamase was 96.1%, 92% and 100%, respectively. Overall ceftazidime/avibactam susceptibility was 99%. For those isolates harbouring an OXA-48-like carbapenemase (without CTX-M but possibly some native AmpC activity) the cefepime/enmetazobactam susceptibility was 100%. Whilst the percentage cefepime/enmetazobactam susceptibility in isolates containing OXA-48-like carbapenemase and a CTX-M enzymes was 89.1%. For *Escherichia coli*, *Klebsiella pneumoniae* and *Enterobacter cloacae*, cefepime/enmetazobactam susceptibility was 98.8%, 87.1% and 83.3%, respectively. For all other species susceptibility was 100%. Only 12 (5.9%) isolates exhibited cefepime/enmetazobactam resistance, with MICs ranging from 8 mg/L to >128 mg/L. However, seven of these isolates had MICs just above the breakpoint (8 mg/L). Resistance mechanisms present were CTX-M ESBL or AmpC alongside efflux.

**Conclusions:**

Cefepime/enmetazobactam has good activity against third generation cephalosporin and carbapenem resistant Enterobacterales. The agent has excellent activity against all third generation cephalosporin resistance mechanisms (native and plasmid-mediated AmpC and ESBL) as well as carbapenem resistance due to OXA-48 like carbapenemases and efflux.

